# TRAF molecules in cell signaling and in human diseases

**DOI:** 10.1186/1750-2187-8-7

**Published:** 2013-06-13

**Authors:** Ping Xie

**Affiliations:** 1 Department of Cell Biology and Neuroscience, Rutgers University, 604 Allison Road, Nelson Labs Room B336, Piscataway, New Jersey 08854

**Keywords:** TRAFs, TNF-Rs, TLRs, NLRs, RLRs, E3 Ubiquitin ligases, DUBs

## Abstract

The tumor necrosis factor receptor (TNF-R)-associated factor (TRAF) family of intracellular proteins were originally identified as signaling adaptors that bind directly to the cytoplasmic regions of receptors of the TNF-R superfamily. The past decade has witnessed rapid expansion of receptor families identified to employ TRAFs for signaling. These include Toll-like receptors (TLRs), NOD-like receptors (NLRs), RIG-I-like receptors (RLRs), T cell receptor, IL-1 receptor family, IL-17 receptors, IFN receptors and TGFβ receptors. In addition to their role as adaptor proteins, most TRAFs also act as E3 ubiquitin ligases to activate downstream signaling events. TRAF-dependent signaling pathways typically lead to the activation of nuclear factor-κBs (NF-κBs), mitogen-activated protein kinases (MAPKs), or interferon-regulatory factors (IRFs). Compelling evidence obtained from germ-line and cell-specific TRAF-deficient mice demonstrates that each TRAF plays indispensable and non-redundant physiological roles, regulating innate and adaptive immunity, embryonic development, tissue homeostasis, stress response, and bone metabolism. Notably, mounting evidence implicates TRAFs in the pathogenesis of human diseases such as cancers and autoimmune diseases, which has sparked new appreciation and interest in TRAF research. This review presents an overview of the current knowledge of TRAFs, with an emphasis on recent findings concerning TRAF molecules in signaling and in human diseases.

## Background

**Figure 1:**
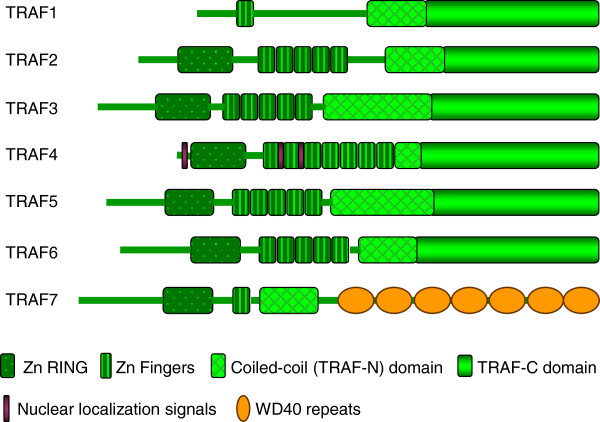
**Domain structure of the seven TRAF proteins.** Symbols for different domains are shown, including zinc RING (Zn RING), zinc fingers (Zn Fingers), coiled-coil (TRAF-N) domain, TRAF-C domain, nuclear localization signals, and WD40 repeats.

The tumor necrosis factor receptor (TNF-R)-associated factor (TRAF) family of intracellular proteins were originally identified as signaling adaptors that bind directly to the cytoplasmic regions of receptors of the TNF-R superfamily [1-3]. There are six known members of the TRAF family (TRAF1 to 6) in mammals. Although a novel protein was named TRAF7 [4], this claim is controversial as the protein does not have the TRAF homology domain that defines the TRAF family (Figure 1). The distinctive feature of all TRAF proteins is a C-terminal TRAF domain, which is composed of an N-terminal coiled-coil region (TRAF-N) and a C-terminal β-sandwich (TRAF-C) [2,3,5]. The TRAF domain mediates protein-protein interactions, including TRAF oligomerization as well as interactions with upstream regulators and downstream effectors [2,3,5]. For example, the eight-stranded β-sandwich structure of the TRAF-C domain mediates the interaction with receptors, and the minor structural differences in this domain among TRAFs (as revealed by X-ray crystallography) define the specificity of each TRAF binding to various receptors [6,7]. Therefore, one important role of TRAFs is to serve as adaptor proteins in the assembly of receptor-associated signaling complexes, linking upstream receptors to downstream effector enzymes. Most TRAFs, with the exception of TRAF1, contain an N-terminal RING finger domain, followed by a variable number of zinc fingers [2,3,8]. The RING finger is found in many E3 ubiquitin ligases and comprises the core of the ubiquitin ligase catalytic domain. Indeed, increasing evidence indicates that in addition to their role as adaptor proteins, TRAFs (including TRAF2, 3, 5 and 6) also act as E3 ubiquitin ligases [3,8,9]. Thus, TRAFs function as both adaptor proteins and E3 ubiquitin ligases to regulate signaling.

The past decade has witnessed rapid expansion of receptor families identified to employ TRAF proteins for signaling. In addition to the TNF-R superfamily, TRAFs are now recognized as signal transducers of a wide variety of other receptor families, including innate immune receptors, adaptive immune receptors, cytokine receptors, and C-type lectin receptors [9-14]. For example, three major families of pattern recognition receptors (PRRs) of the innate immune system recruit TRAFs via additional adaptor proteins: Toll-like receptors (TLRs) via MyD88 or TRIF, nucleotide binding-oligomerization domain (NOD)-like receptors (NLRs) via RIP2, and retinoic acid-inducible gene I (RIG-I)-like receptors (RLRs) via MAVS [10,15,16]. TRAF-dependent signaling pathways typically lead to the activation of nuclear factor-κBs (NF-κB1 and NF-κB2), mitogen-activated protein kinases (MAPKs), or interferon-regulatory factors (IRFs). Acting alone or in combination, TRAFs are highly versatile regulators that control diverse cellular processes, including survival, proliferation, differentiation, activation, cytokine production, and autophagy [3,9,16,17].

Despite the similarities in the signaling pathways activated by different TRAF proteins, each TRAF appears to play obligatory and non-redundant physiological roles. Germ-line and conditional knockout mice have been instrumental in revealing the overlapping yet distinct roles of different TRAFs in whole animals. Compelling evidence from these studies demonstrates that TRAFs critically regulate a plethora of physiological processes, including innate and adaptive immunity, embryonic development, tissue homeostasis, stress response, and bone metabolism [3,18,19]. The pivotal roles of TRAFs in host immunity are further highlighted by the discoveries that pathogens adopt deliberate strategies to subvert TRAF functions [20,21]. An emerging paradigm of TRAF functions is that alterations in TRAFs may contribute to the pathogenesis of important human diseases, including cancers, autoimmune diseases and immunodeficiencies [18,22,23]. This has sparked new appreciation and interest in TRAF research during the past few years. Here I attempt to provide an overview of the current knowledge of TRAFs, with an emphasis on recent advances in understanding TRAFs in receptor signaling and in human diseases as well as recent insights into the regulatory mechanisms of TRAF ubiquitination.

### TRAFs in signaling by the TNF-R superfamily

Receptors of the TNF-R superfamily have wide tissue distribution and regulate diverse biological functions, including immune responses, inflammation, lymphoid organ and brain development, osteoclastogenesis, and tissue homeostasis [3,24-26]. Structurally, these receptors are characterized by the presence of conserved cysteine-rich domains (CRDs) in their extracellular region that are responsible for the binding of their ligands of the TNF superfamily. Based on the intracellular structures, the TNF-R superfamily is categorized into two main groups. The first group of receptors, termed death receptors, contain a death domain in the intracellular region. The second group, also the majority of the TNF-R superfamily, do not have a death domain but contain TRAF-interacting motifs (TIMs) in their intracellular region [3,24,26]. TRAF2, 3 and 5 usually have overlapping binding motifs, whereas TRAF6 has a distinct interacting motif on these receptors [3,27].

Receptors of this family do not have kinase activity and depend on the binding of adaptor proteins to assemble signaling complexes to activate downstream pathways [3,24,26]. Signaling by death receptors mainly relies on adaptor proteins containing a death domain, such as TRADD or FADD, thereby culminating in caspase activation and cell apoptosis. In contrast, signaling by the TIM-containing receptors is mediated primarily, albeit not exclusively, via TRAFs [3,24,26]. These include TRAFs that can interact with the receptors either directly through TIMs or indirectly through other TRAFs or adaptor proteins (Table 1). Binding of TRAFs to TNF-Rs typically induces signaling cascades leading to the activation of NF-κB and MAPKs, including ERK, p38 and JNK, and ultimately regulates cell survival or functionality depending on the cell type and the context [3,24,26]. Notably, TRAF2 and TRAF5 can also modulate signaling by death receptors through association with TRADD, FADD or RIP1 (Table 1). Most TRAF-dependent receptors of this family trigger the canonical NF-κB pathway (RelA/p50, NF-κB1). In contrast, the alternative NF-κB pathway (RelB/p52, NF-κB2) is activated by a subset of TNF-Rs, including CD40, BAFF-R, the lymphotoxin-β receptor (LTβR), 4-1BB, and Fn14 [28-32]. Interestingly, however, unlike CD40 or BAFF-R, TWEAK-induced Fn14 signaling promotes NF-κB2 activation through a distinct mechanism that induces lysosomal degradation of cIAP1-TRAF2 in a cIAP1-dependent manner [33]. The distinct TWEAK/Fn14 paradigm is covered in detail in a recent review by Silke and Brink [32].

**Table 1:** TRAFs directly and indirectly employed by the TNF-R superfamily

**Receptors**	**TRAFs**	**References**
**Receptors containing TRAF-interacting motifs**		
TNF-R2	TRAF2	[26,34]
	TRAF1, 3 via TRAF2	[26]
CD40	TRAF2, 3, 5, 6	[27,28,35]
	TRAF1 via TRAF2	[28]
BAFF-R	TRAF3, 6	[36-38]
	TRAF2 via TRAF3	[28]
BCMA	TRAF1, 2, 3, 5, 6	[39,40]
TACI	TRAF2, 3, 5, 6	[2,28,41]
LTβR	TRAF2, 3, 5	[29,42-47]
CD27	TRAF2, 3, 5	[1,48,49]
CD30	TRAF1, 2, 3, 5	[1,2]
4-1BB	TRAF1, 2, 3	[1,2]
OX40	TRAF1, 2, 3, 5, 6	[1,2,50-52]
GITR	TRAF1, 2, 3, 4, 5	[1,2,53,54]
RANK	TRAF1, 2, 3, 5, 6	[1,2,55,56]
HVEM	TRAF1, 2, 3, 5	[1,2,57]
Troy	TRAF2, 5, 6	[58]
XEDAR	TRAF3, 6	[59]
Fn14	TRAF2, 6	[33,60]
**Death receptors**		
TNF-R1	TRAF2 via TRADD	[61-63]
	TRAF5 via RIP1	[64]
p75NTR	TRAF1, 2, 3, 4, 5, 6	[2,65,66]
EDAR	TRAF1, 3, 6	[63]
FAS	TRAF2 via FADD	[63]
DR3	TRAF2 via TRADD	[63]
DR6	TRAF2 via TRADD	[63]
TRAIL-R1	TRAF2 via RIP1	[67]

**Figure 2:**
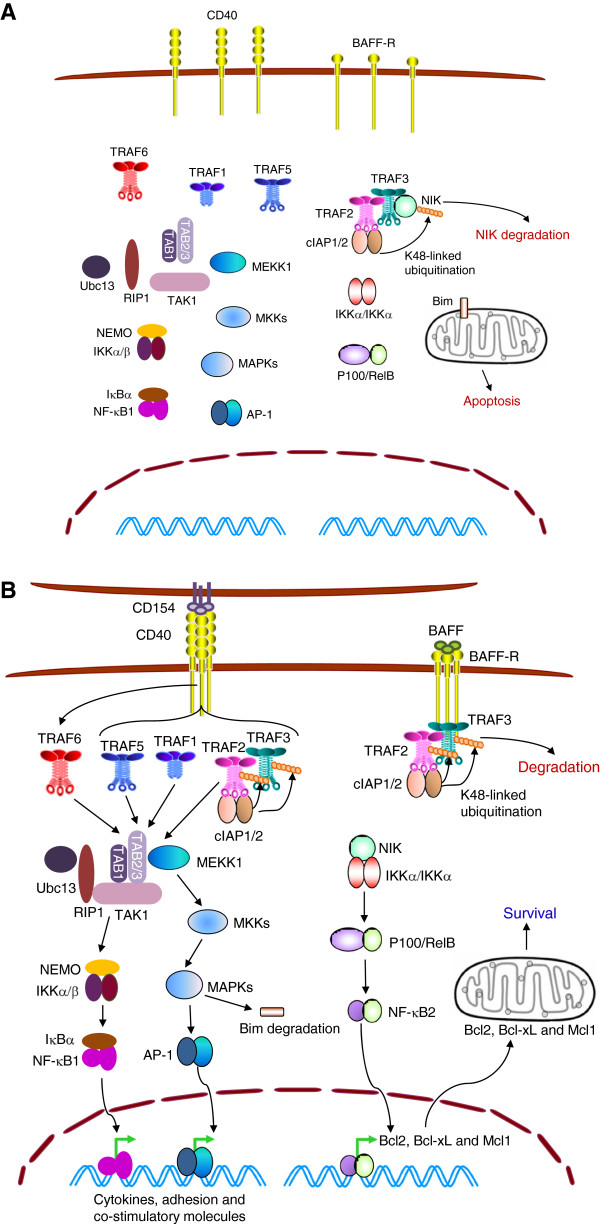
**TRAFs in BAFF-R and CD40 signaling pathways in B lymphocytes.** (**A**) In the absence of stimulation, TRAF3 and TRAF2 promote B cell apoptosis. TRAF3 and TRAF2 constitutively form a complex with cIAP1/2 and NIK, target NIK for K48-linked polyubiquitination and degradation, thereby inhibiting NF-κB2 activation in B cells. (**B**) BAFF-R and CD40 signaling pathways. Upon ligand engagement, BAFF-R or CD40 recruits TRAF3-TRAF2-cIAP1/2 to membrane rafts, thus allowing NIK accumulation and NF-κB2 activation, leading to B cell survival. In addition, TRAF1, 2, 5 and 6 mediate CD40-induced activation of NF-κB1 and MAPKs.

Using CD40 and BAFF-R as examples, here I briefly summarize recent advances in understanding how TRAFs regulate the two NF-κB pathways and activation of MAPKs (Figure 2). In the absence of stimulation, TRAF3 constitutively binds to NIK (the upstream kinase of the NF-κB2 pathway) and TRAF2 (which associates with cIAP1/2). In this complex, cIAP1/2 induces K48-linked polyubiquitination of NIK, and thus targets NIK for proteasomal degradation and inhibits NF-κB2 activation [37,68-71]. Following BAFF or CD154 stimulation, trimerized BAFF-R or CD40 recruits TRAF3, TRAF2, cIAP1/2 and MALT1 to membrane signaling rafts, releasing NIK from the TRAF3/TRAF2/cIAP1/2 complex [37,72-74]. NIK protein is accumulated in the cytoplasm, induces the activation of IKKα and NF-κB2, and eventually up-regulates the expression of anti-apoptotic proteins of the Bcl-2 family (such as Bcl-2, Bcl-xL, and Mcl-1) to induce cell survival [28]. In the receptor signaling complex, TRAF2 induces K63-linked polyubiquitination of cIAP1/2, which is subsequently activated to catalyze K48-linked polyubiquitination and degradation of TRAF3 and TRAF2 [37,72,74,75]. Following CD40 activation, many other signaling proteins (including TRAF5, TRAF6, TRAF1, Ubc13, MEKK1, TAK1 and NEMO) are also recruited to the cytoplasmic domain of the receptor, and the K63-specific ubiquitin ligase activity of TRAF2 and TRAF6 is rapidly stimulated [27,72,75]. These proteins form several separate multiprotein signaling complexes, which result in the phosphorylation and activation of MEKK1 and TAK1. Activated MEKK1 and TAK1 and their associated protein complexes are subsequently released from the receptor into the cytoplasm to activate MAPKs and NF-κB1, which eventually mediate the effector functions of CD40 [35,72]. Interestingly, the releasing step of MEKK1 and TAK1 is inhibited by TRAF3 via a yet unknown mechanism, but promoted by cIAP1/2-catalyzed K48-linked polyubiquitination and proteasomal degradation of TRAF3 [72,74,75]. In response to BAFF stimulation, a signaling pathway of c-Raf-MEK-ERK-dependent phosphorylation and down-regulation of the pro-apoptotic protein Bim also contributes to B cell survival [76]. In light of the evidence that TRAF1 mediates 4-1BB-induced ERK-dependent phosphorylation and down-regulation of Bim to promote T cell survival [77-79], it would be interesting to investigate the role of TRAF1 in BAFF-induced Bim down-regulation in B cells. Collectively, the above evidence indicates that TRAFs are critical regulators of signaling by the TNF-R superfamily.

### TRAFs in TLR signaling

Toll-like receptors (TLRs), the best-studied family of PRRs, recognize conserved structures termed pathogen-associated molecular patterns (PAMPs) of diverse invading microbes, including Gram-positive and -negative bacteria, DNA and RNA viruses, fungi, protozoa, and parasites. They also detect endogenous molecules released from damaged or inflamed self-tissues, referred to as damage-associated molecular patterns (DAMPs) [80-82]. Upon sensing these molecules, TLR signaling induces the production of pro-inflammatory cytokines (such as TNFα, IL-1, IL-6, and IL-12), type I interferons (IFNα and IFNβ), chemokines, antimicrobial enzymes, and other inflammatory mediators. These provoke acute inflammatory responses as well as phagocytosis and autophagy, which represent the first line of innate immunity against pathogens [17,83,84]. TLR signaling also serves to prime the subsequent adaptive immune responses by up-regulating adhesion molecules and co-stimulatory molecules (such as CD40, CD80, and CD86) on antigen presenting cells [85,86].

TLRs (TLR1, 2, 4–6, 10) that sense lipids or proteins are located on the cell membrane, while those (TLR3, 7, 8, 9) that recognize nucleic acids are resided in intracellular endosomes [8,87]. Each TLR consists of an ectodomain containing leucine-rich repeats (LRR) that mediate sensing of PAMPs or DAMPs, and a cytoplasmic Toll/IL-1 receptor (TIR) domain that mediates downstream signal transduction. Ligand-induced TLR dimerization or oligomerization recruits TIR domain-containing adaptor proteins through TIR-TIR interactions, including MyD88, TRIF, Mal and TRAM [83,88,89]. MyD88 is employed by all TLRs except TLR3. TRIF is only used by TLR3 and endocytosed TLR4. Mal (also known as TIRAP) facilitates the recruitment of MyD88 to TLR4, while TRAM acts as a bridging adaptor between TRIF and endocytosed TLR4. Collectively, two general pathways are used by TLRs: MyD88-dependent (all TLRs except TLR3) and TRIF-dependent (TLR3 and TLR4) pathways. Both pathways initiate complex signaling cascades of phosphorylation and ubiquitination events, which culminate in the activation of transcription factors, including NF-κB, IRFs, and AP-1 family members, leading to innate immune responses [83,88,89].

**Figure 3:**
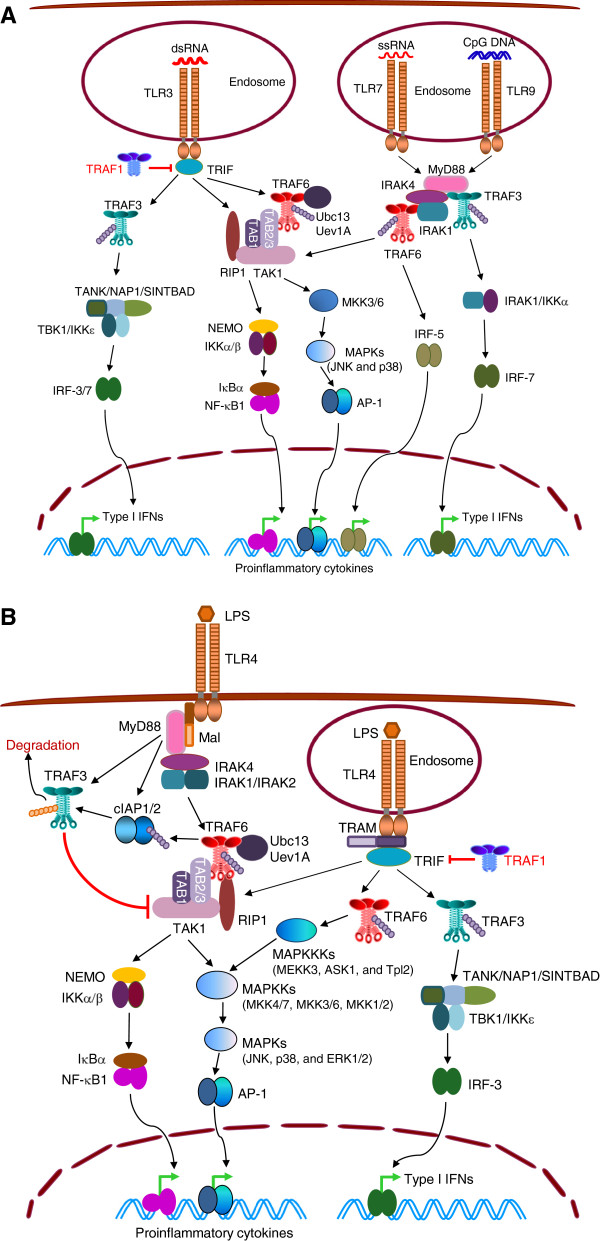
**TRAFs in signaling by TLRs.** (**A**) TLR3, 7 and 9 signaling pathways. Upon ligand binding in endosomes, TLR3 recruits TRAF3 and TRAF6 via TRIF, while TLR7 and TLR9 recruit TRAF3 and TRAF6 via MyD88-IRAK1. (**B**) TLR4 signaling pathways. Upon LPS engagement on the plasma membrane, TLR4 recruits TRAF6 and TRAF3 via MyD88-IRAK1. Internalized TLR4 recruits TRAF3 and TRAF6 to endosomes via TRIF. TRAF6 mediates MyD88- and TRIF-induced activation of NF-κB1 and MAPKs, while TRAF3 mediates MyD88- or TRIF-induced activation of IRF-3/7 in signaling by TLRs. In contrast, TRAF1 inhibits TRIF signaling.

TRAF6 mediates both MyD88-dependent and TRIF-dependent activation of NF-κB and AP-1 (Figure 3). In MyD88-dependent TLR signaling, TRAF6 is recruited to MyD88-activated IRAK1/2, and oligomerization of TRAF6 stimulates its E3 ubiquitin ligase activity. In coordination with the E2 complex Uev1A:Ubc13, TRAF6 catalyzes the attachment of K63-linked polyubiquitin chains onto its substrates, including itself and NEMO [8,89,90], and synthesis of free, unanchored K63-polyubiquitin chains [91]. Ubiquitinated TRAF6 serves as a signaling scaffold to recruit TAK1 via TAB2/3. TRAF6-generated free K63-polyubiquitin chains also bind to TAB2/3 to activate TAK1, and bind to NEMO to activate IKKα/β in the receptor complex. This ultimately results in MyD88-dependent activation of NF-κB [8,82,90,91]. The TAK1 signaling complex, including TRAF6-TAB2/3-TAB1-TAK1, is subsequently dissociated from the receptor and released into the cytosol, where TAK1 activates MAPK cascades, leading to activation of AP-1. Similar to CD40 signaling, the release of the TAK1 signaling complex from TLR4 is inhibited by TRAF3, which is recruited to TLR4 by MyD88 and IRAK1. However, TRAF6 catalyzes K63-linked polyubiquitination of cIAP1/2, which is also recruited by MyD88 and IRAK1. Activated cIAP1/2 promotes K48-linked polyubiquitination and degradation of TRAF3, allowing activation of MAPKs [92]. In TRIF-dependent TLR signaling, TRIF directly recruits TRAF6 and RIP1, which work cooperatively to activate TAK1, eventually leading to activation of NF-κB and AP-1 [8,82,90]. Interestingly, in response to engagement of TLR1, 2 or 4, TRAF6 is also translocated to mitochondria, where it ubiquitinates evolutionarily conserved signaling intermediate in Toll pathways (ECSIT), resulting in increased reactive oxygen species (ROS) generation and bacteria killing [93]. Notably, TRAF6 is also necessary for IRF7 activation and type I IFN production induced by TLR7 and TLR9 in plasmacytoid dendritic cells (pDCs) [94].

TRAF3 is required for both MyD88-dependent and TRIF-dependent activation of IRF3 and IRF7, and thus production of type I IFNs [95,96], a class of cytokines with potent antiviral and antibacterial activities. In MyD88-dependent signaling downstream of TLR7 and TLR9, TRAF3 is recruited to MyD88 and IRAK1. Activated TRAF3 catalyzes its K63-linked auto-ubiquitination, and assembles a signaling complex with MyD88, IRAK4, IRAK1, IKKα and IRF7. Within this complex, IRF7 is phosphorylated and activated by IRAK1 and IKKα to induce the production of type I IFNs [8,82,86]. In TRIF-dependent signaling downstream of TLR3 and TLR4, TRAF3 interacts with oligomerized TRIF, and activated TRAF3 recruits TBK1 and IKKϵ through NAP1 and TANK. In this signaling complex, TRAF3, in cooperation with Ubc13 and/or Ubc5, catalyzes K63-polyubiquitination of TRAF3 itself, TBK1 and IKKϵ, which facilitates the phosphorylation of IRF3 and IRF7. The phosphorylated IRF3 and IRF7, in turn, form homodimers or heterodimers, translocate into the nucleus and induce the expression of type I IFNs as well as IFN-inducible gene [8,82,86] (Figure 3).

Interestingly, TRAF1 was also identified as a TRIF-interacting protein in yeast two-hybrid screens. Overexpression of TRAF1 inhibits TRIF- and TLR3-mediated activation of NF-κB and expression of IFN-β, suggesting that TRAF1 inhibits TRIF-dependent signaling [83,97,98]. Similarly, TRAF4 physically interacts with and functionally counteracts TRAF6 and TRIF in TLR signaling [99]. Taken together, recent advances indicate that TRAF6, TRAF3, TRAF1 and TRAF4 play critical and largely distinct roles in signaling by TLRs.

### TRAFs in NLR signaling

NOD-like receptors (NLRs) are a family of cytosolic sensors of PAMPs and DAMPs, and are functionally analogous to TLRs [100-102]. Each NLR appears to be activated by multiple agonists. However, in many cases, evidence of direct interaction between NLRs and PAMPs/DAMPs is lacking [103,104]. Effector functions of NLRs include secretion of pro-inflammatory cytokines, chemokines, anti-microbial peptides and type I IFNs, generation of ROS, autophagy, antigen processing, and expression of MHC class II on antigen presenting cells. These responses induce innate immune clearance of the pathogen, and also tailor the adaptive immune system to fight the infection [100-102]. NLRs are characterized by a central NOD domain that mediates nucleotide-binding and oligomerization, and the C-terminal LRRs that possibly mediate ligand detection. In addition, they contain N-terminal effector domains, such as caspase recruitment domains (CARD), pyrin domains (PYD), baculovirus inhibitor of apoptosis repeat (BIR) domains, or an acidic transactivation domain, which recruit downstream signal transduction molecules after ligand sensing [100,101,104]. One well-studied pathway of several NLRs, including NLRP3, NLRP1, NLRP6, and NLRC4, is the assembly of multi-protein complexes called ‘inflammasomes’, which contain caspase-1 and apoptosis-associated speck-like protein containing a CARD (ASC). Inflammasomes induce proteolytic processing of pro-IL-1β and pro-IL-18 into secretable IL-1β and IL-18, as well as caspase 1-dependent apoptosis termed ‘pyroptosis’ [15,100,101]. The role of TRAF2 in inflammasome signaling has recently been explored, but the published data are contradictory. Labbe *et al.* reported that depletion of TRAF2 by siRNA inhibits inflammasome signaling in HEK293T cells [105]. However, Vince *et al.* found that inflammasome activation is normal in TRAF2^−/−^ bone marrow-derived macrophages (BMDMs) [71]. Potential involvement of other TRAFs in inflammasome signaling remains to be elucidated.

**Figure 4:**
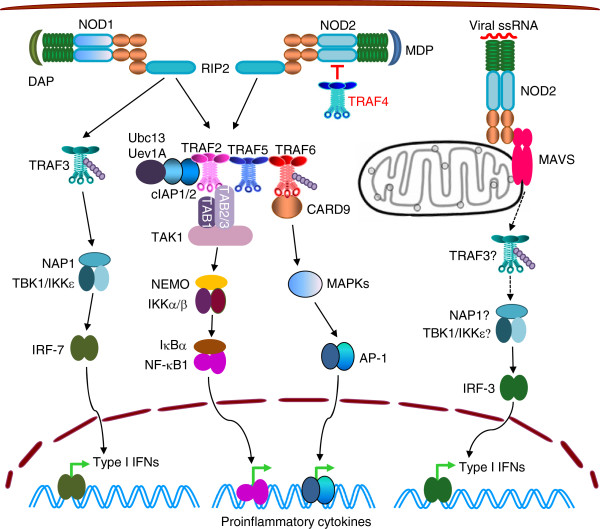
**TRAFs in signaling by NOD1 and NOD2.** Upon DAP engagement, NOD1 recruits TRAF2, TRAF5, TRAF6 and TRAF3 via RIP2. TRAF2, 5 and 6 mediate NOD1-induced activation of NF-κB1 and MAPKs, while TRAF3 mediates NOD1-induced activation of IRF7. In response to MDP binding, NOD2 also recruits TRAF2, 5 and 6 via RIP2, and thus induces activation of NF-κB1 and MAPKs. When engaged by viral ssRNA, NOD2 binds to MAVS on mitochondria and induces IRF3 activation and Type I IFN production, which is likely mediated by TRAF3.

TRAF2, TRAF5, and TRAF6 are required for NF-κB and MAPK activation induced by NOD1 and NOD2 (Figure 4), the founding members of the NLR family [15,102,106]. Upon detection of *meso*-diaminopimelic acid (DAP) by NOD1 or muramyl dipeptide (MDP) by NOD2 at the vicinity of plasma membranes, oligomerization of NOD1 or NOD2 recruits the dual specificity kinase RIP2 (also called RICK) via a homotypic CARD-CARD interactions [101-103]. Activated RIP2 induces the formation of the signaling complex containing TRAF2, TRAF5, TRAF6, TRAF4, CARD9, cIAP1/2, and Ubc13/Uev1A. In this complex, cIAP1/2, in coordination with Ubc13/Uev1A, catalyze K63-linked polyubiquitination of RIP2, which further recruits TAB2/3-TAB1-TAK1 and NEMO-IKKα/β, leading to NF-κB activation [15,100,107-109]. Interestingly, a recent study by Damgaard *et al.* demonstrated that XIAP is also recruited to the NOD2 signaling complex, in which XIAP primarily conjugates ubiquitin chains on RIP2 that are linked through lysine residues other than K63 and K48 [110]. Thus, XIAP, together with cIAP1/2, constitutes the major ubiquitin ligase activity that ubiquitinates RIP2 in NOD2 signaling, and cIAP1/2 appear to be rate limiting only when XIAP is not present [110]. It has been shown that TRAF2 and TRAF5 are required for NOD-induced NF-κB activation, while TRAF6, CARD9, and ITCH are important for p38 and JNK activation in NOD signaling [15,111,112]. However, the exact mechanism of how these occur is still unknown. Interestingly, TRAF4 is identified as a key negative regulator of NOD2 signaling. TRAF4 binds directly to NOD2 in an agonist-dependent manner, and inhibits NOD2-induced NF-κB activation and bacterial killing [109]. This inhibitory effect of TRAF4 requires its phosphorylation at Ser426 by IKKα, which is also recruited to the NOD2 signaling complex [113].

TRAF3 mediates type I IFN production induced by NOD1 [114], and presumably also that induced by NOD2 (Figure 4). NOD1 and NOD2 induce type I IFN production through distinct mechanisms. Upon sensing DAP, oligomerization of NOD1 recruits TRAF3 via RIP2. TRAF3 in turn activates TBK1 and IKKϵ, leading to subsequent activation of IRF7 and type I IFN production in epithelial cells [100,102,114]. In contrast, NOD2 induces type I IFN production only in response to viral ssRNA, but not in response to MDP, via a RIP2-independent pathway [102,115]. Following the detection of viral ssRNA, NOD2 engages a signaling complex containing MAVS on mitochondria, which induces IRF3 activation and type I IFN production [115]. TRAF3 has been shown to directly interact with MAVS to mediate RLR-induced type I IFN production [116]. It is thus speculated that TRAF3 may similarly activate TBK1 and IKKϵ in NOD2-MAVS signaling, but this awaits experimental investigation.

Interestingly, TRAF3 and TRAF6 are involved in the cross-talk between several NLRs and TLRs or RLRs. TRAF3 regulates NLRP12-mediated suppression of TLR-driven NF-κB activation, as NLRP12 interacts with both NIK and TRAF3 [117]. TRAF6 interacts with NLRX1, which negatively regulates NF-κB activation induced by RIG-I or TLR4 [118,119]. Similarly, NLRC3 also inhibits TLR-induced NF-κB activation by interacting with TRAF6 and reducing K63-linked polyubiquitination of TRAF6 [120].

### TRAFs in RLR signaling

**Figure 5:**
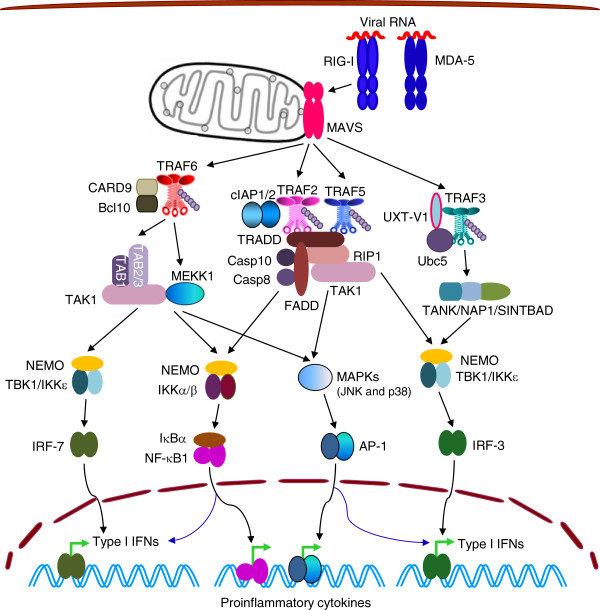
**TRAFs in signaling by RIG-I.** Upon ligand binding, RIG-I recruits TRAF3, TRAF6, TRAF2 and TRAF5 to mitochondria via MAVS. TRAF3 mediates RIG-I-induced IRF3 but not NF-κB1 activation. TRAF6 mediates RIG-I-induced IRF7 activation and also contributes to activation of NF-κB1, JNK, and p38. TRAF2 is important for p38 activation, and both TRAF2 and TRAF5 also contribute to activation of IRF3 and NF-κB1 in RIG-I signaling.

RIG-I like receptors (RLRs), including RIG-I, MDA5, and LGP2, are a family of cytosolic RNA helicases that detect viral RNA PAMPs accumulated during viral infection or replication. RLRs are indispensable for antiviral responses in most cell types except pDCs [116,121,122]. RIG-I/MDA5 signaling rapidly elicits the production of type I and type III IFNs and proinflammatory cytokines. RIG-I and MDA5 exhibit different ligand specificity and respond to different viruses, whereas LGP2 facilitates or antagonizes recognition of viral RNA by MDA5 and RIG-I [116,121,122]. RLRs are structurally characterized by a central DExD/H box RNA helicase domain involved in RNA binding and ATPase function, and a carboxyl-terminal domain (CTD) that contains a positively charged RNA binding pocket. RIG-I and MDA5, but not LGP2, also possess two N-terminal CARDs that are required to trigger downstream signaling [116,123,124]. Upon detection of RNA PAMPs, RIG-I/MDA-5 undergoes conformational change that leads to dimerization and association with the mitochondrial antiviral signaling adaptor (MAVS, also called IPS-1, VISA, or Cardif) through homotypic CARD-CARD interactions [116,121,122]. MAVS consists of an N-terminal CARD domain, a central proline-rich region (PRR), several TRAF-interacting motifs, and a C-terminal transmembrane domain, which anchors the protein on the outer membranes of mitochondria. Dimerization of MAVS directly recruits TRAF2 [125], TRAF3 [126], TRAF5, TRAF6 [127], CARD9 and TRADD, which serve as a platform to assemble signaling complexes at mitochondrial outer membranes [123,124,128,129]. These signaling complexes contain players that are further recruited by TRAFs or TRADD, including cIAP1/2, TANK-NAP1-SINTBAD, TBK1-IKKϵ, NEMO, IKKα/IKKβ, TAB2/3-TAB1-TAK1, MEKK1, Bcl10, and RIP1-FADD-Casp8-Casp10. RIG-I/MDA5 signaling cascades culminate in the phosphorylation and activation of IRF3, IRF7, NF-κB and AP-1, which work cooperatively to induce the expression of IFNs and proinflammatory cytokines (Figure 5) [123,124,128,129].

TRAF3 is essential for RLR-induced IRF3 but not NF-κB activation, and TRAF3 deficiency results in impaired type I IFN induction in response to RNA virus infection [126]. MAVS has a TRAF3-interacting motif in the C-terminus that is verified by crystallography [130,131], and Tyr9 phosphorylation on MAVS also facilitates the recruitment of TRAF3 [132]. Additionally, TRAF3-MAVS interaction requires the assistance of another TRAF3-interacting protein, UXT-V1 [133]. Following its recruitment to MAVS and in conjunction with Ubc5, TRAF3 undergoes K63-linked auto-ubiquitination, which enhances its ability to bind to NEMO and TANK-NAP1-SINTBAD, thus allowing the recruitment and activation of TBK1 and IKKϵ [116,128,134-137]. Interestingly, a recent study shows that linear ubiquitination of NEMO switches it from a positive to a negative regulator of RIG-I signaling, as linear ubiquitinated NEMO associates with TRAF3 but disrupts the MAVS-TRAF3 complex [138]. The NEMO-like adaptor proteins TANK, NAP1, and SINTBAD are constitutively bound to both TBK1 and IKKϵ [135]. Autoubiquitinated TRAF3 activates TBK1 and IKKϵ to induce the phosphorylation, dimerization and nuclear translocation of IRF3, which triggers the production of type I IFNs [16,128,129].

Depletion of either TRAF2 or TRAF5 leads to reduced IRF3 and NF-κB activation upon RIG-I stimulation, and TRAF2 and TAK1 are important for p38 activation [125,128,139,140]. Biochemical studies revealed TRAF2 and TRAF5 interaction motifs in the C-terminal region of MAVS. Upon RIG-I signaling, interaction of TRAF5 with MAVS induces K63-linked TRAF5 auto-ubiquitination and subsequent NEMO-dependent activation of IRF3 and NF-κB [139]. Similarly, activation of p38 by RIG-I proceeds via a TRAF2-TAK1-dependent pathway. The p38 activation in turn stimulates the production of IFNs and IL-12 [125]. Nonetheless, details of TRAF2- or TRAF5- signaling pathways downstream of MAVS remain to be elucidated.

TRAF6 is required for RLR-induced IRF7 activation and also contributes to activation of NF-κB, JNK, and p38 by directly interacting with MAVS, which has two TRAF6-interacting motifs [127,141]. Activation of IRF7 after viral infection resembles IRF3 activation, and involves the direct phosphorylation of IRF7 by TBK1 and IKKϵ. However, activation of IRF7 but not IRF3 is impaired in TRAF6^−/−^ fibroblasts, and TRAF6 mediates IRF7 ubiquitination [141,142]. Thus, MAVS-induced IRF7 activation is transduced through a unique TRAF6-dependent pathway. Uncoupling IRF3 from the IRF7 activation pathway might be a way of avoiding their simultaneous inhibition by virus-encoded inhibitory proteins [128]. TRAF6 and MEKK1 are also important for RLR-induced activation of NF-κB and MAPKs [127,141]. Interestingly, RIG-I-MAVS-TRAF6 signaling leads to IKKβ-mediated phosphorylation of p65 at ser536, which is under the control of the NADPH oxidase NOX2 [143].

Notably, cIAP1 and cIAP2 are also recruited to MAVS, and mediate K48- and K63- linked polyubiquitination of TRAF3 and TRAF6 in response to viral infections [144]. However, the kinetics of these two types of ubiquitination on TRAF3 and TRAF6 is still unclear. Interestingly, viruses also induce IRF3-dependent apoptosis in infected cells, which require the presence of RIG-I, MAVS, TRAF3, TRAF2, TRAF6 and TBK1, as demonstrated by studies using genetically defective mouse and human cell lines [140]. Apoptosis is triggered by direct interaction of IRF3, through a newly identified BH3 domain, with the pro-apoptotic protein Bax. Co-translocation of IRF3 and Bax to mitochondria results in the induction of mitochondria-dependent apoptosis, and transcriptionally inactive IRF3 mutants could efficiently mediate apoptosis [140]. Although why TRAF3, TRAF2 and TRAF6 are all required for IRF3-induced apoptosis awaits further clarification, it appears that these TRAF molecules cooperate in this process.

### TRAFs in cytokine receptor signaling

It was initially recognized that TRAF6 is utilized for signaling by the IL-1R family (IL-1R, IL-18R and IL-33R), which also contain TIR domains found in TLRs [23,145,146]. However, recent evidence indicates that TRAFs also directly regulate signaling by a variety of other cytokine receptors, including receptors for the proinflammatory IL-17 family, anti-viral IFNs, anti-inflammatory TGFβ, and the T cell cytokine IL-2.

#### IL-17 receptors

**Figure 6:**
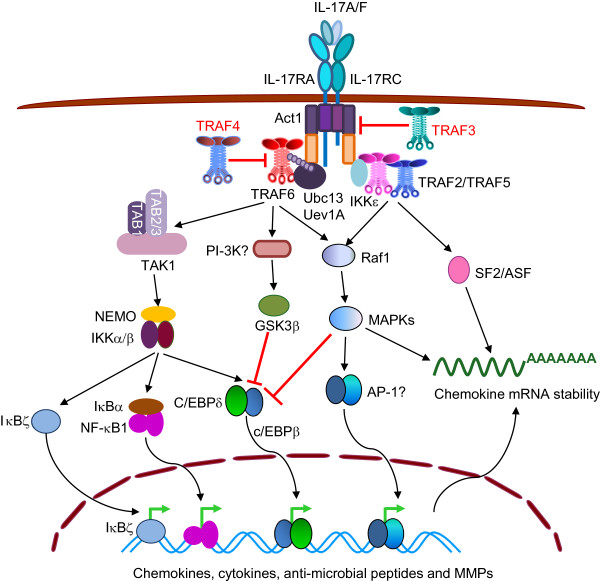
**TRAFs in signaling by IL-17R.** Upon ligand binding, heteromeric IL-17RA and IL-17RC recruit TRAF6, TRAF2 and TRAF5 via Act1. TRAF6 mediates IL-17-induced activation of NF-κB1, IkBζ, C/EBPδ and C/EBPβ. TRAF2 and TRAF5 transduce the IL-17 signals to stabilize mRNA transcripts of chemokines and cytokines by recruiting SF2 and by inducing activation of MAPKs. In contrast, TRAF3 and TRAF4 inhibit IL-17 signaling. TRAF3 interacts with IL-17RA and IL-17RC and thus interferes with the recruitment of Act1 by IL-17Rs, while TRAF4 binds to Act1 and interrupts the recruitment of TRAF6 by Act1.

The IL-17 family are important in host defense against bacterial, fungal and helminthic parasite infections [147-149]. The founding member of this family, IL-17, is the defining cytokine of a new T helper cell population termed “Th17”, which contributes significantly to the pathogenesis of multiple autoimmune and inflammatory diseases [150,151]. Signature target genes of IL-17 include chemokines, proinflammatory cytokines, inflammatory mediators, anti-microbial peptide, and matrix metalloproteases (MMPs) [147-149]. IL-17 (A/F) signals through a heteromeric receptor complex formed by IL-17RA and IL-17RC. IL-17Rs have two extracellular fibronectin III-like domains and a cytoplasmic SEF/IL-17R (SEFIR) domain [149,150]. Ligand-induced association of IL-17RA and IL-17RC recruits a novel adaptor protein Act1 through SEFIR domain-mediated homotypic interaction. Act1 is a U-box E3 ubiquitin ligase that contains both a SEFIR domain and TIMs, and further recruits TRAF6, TRAF2 and TRAF5 [12,152,153]. In cooperation with Ubc13/Uev1A, Act1 catalyzes K63-linked polyubiquitination of TRAF6, which in turn mediates the ubiquitination of IL-17RA and induces the activation of NF-κB through TAK1 and IKKs. Activated NF-κB further induces the expression of IkBζ, C/EBPδ and C/EBPβ, transcription factors that work in concert with NF-κB to induce the expression of signature target genes of IL-17 [12,147,154-158]. On the other hand, TRAF6 also induces GSK3β activation likely through PI-3K, and ERK1/2 activation likely through Raf1 [12,151,159]. Activated GSK3β and ERK induce dual phosphorylation of C/EBPβ and thereby inhibit its activity [12,151,160]. Thus, TRAF6 is essential for IL-17 signaling (Figure 6).

Interestingly, TRAF2 and TRAF5 transduce the IL-17 signals to stabilize mRNA transcripts of chemokines (such as CXCL1) and cytokines (such as IL-6) by recruiting the splicing factor SF2 (also known as alternative splicing factor, ASF) into the IL-17R-Act1 signaling complex [151,153,161]. The IL-17R-Act1-TRAF2-TRAF5 complex also induces the activation of MAPKs, which further enhance mRNA stability. Notably, formation of this complex requires IKKϵ, an inducible IKK that mediates Act1 phosphorylation at Ser311, adjacent to a putative TRAF-binding motif. Substitution of Ser311 of Act1 with alanine impairs the IL-17-induced Act1-TRAF2-TRAF5 interaction and inflammatory gene expression [161,162]. In contrast, TRAF3 and TRAF4 are negative regulators of IL-17R signaling [12,163,164]. Upon IL-17 stimulation, IL-17RA and IL-17RC directly recruit TRAF3 via a distal C-terminal TRAF3-binding site. The binding of TRAF3 to IL-17Rs interferes with the formation of the activation signaling complex of IL-17R-Act1-TRAF6, resulting in suppression of downstream signaling, including NF-κB and MAPK activation, and production of inflammatory cytokines and chemokines [12,163]. TRAF4 exerts its negative regulation on IL-17 signaling by competing with TRAF6 for the interaction with Act1, as TRAF4 and TRAF6 use the same TIMs on Act1. Indeed, primary epithelial cells derived from TRAF4^−/−^ mice display markedly enhanced IL-17 signaling [164]. Thus, both TRAF3 and TRAF4 restrict IL-17 signaling at receptor proximal steps (Figure 6).

#### IFN receptors

Interferons induce the synthesis of a variety of antiviral proteins that mediate swift innate immune responses to control virus replication and spread, and also shape the adaptive immune response by acting directly on T and B cells [116]. TRAF2 and TRAF6 are recognized as direct signal transducers of IFN receptors. Upon IFN engagement, TRAF2 directly binds to the membrane proximal half of the signal-transducing subunit of the IFN receptor, IFNAR1, and is required for IFN-induced NF-κB2 activation and anti-viral responses [13,165]. Similarly, direct interaction of TRAF6 with the intracellular domain of IFNλR1 regulates NF-κB activation and IFNλR1 stability in response to type III IFNs (IFNλ1, IFNλ2, and IFNλ3) [166]. Whether other TRAFs contribute to the regulation of IFN signaling remains to be determined.

#### TGFβ receptors

The anti-inflammatory cytokine TGFβ binds to type II and type I serine/threonine kinase receptors (TβRII and TβRI). TRAF6 interacts with a consensus TIM present in TβRI [14,167,168]. The TβRI-TRAF6 interaction induces auto-ubiquitination of TRAF6. TβRI kinase activity is required for activation of the canonical Smad pathway, whereas TRAF6 regulates the activation of TAK1 in a receptor kinase-independent manner. Activated TRAF6 mediates K63-linked polyubiquitylation of TAK1 at Lys34 and Lys158, and results in subsequent activation of p38 and JNK, leading to cell apoptosis [14,167,168]. Thus, TRAF6 is specifically required for the Smad-independent activation of JNK and p38 in response to TGFβ. However, in cancer cells, TRAF6-mediated K63-linked polyubiquitination of TβRI also promotes cleavage of TβRI by TNFα converting enzyme (TACE) in a PKCζ-dependent manner. The liberated intracellular domain of TβRI associates with the transcriptional regulator p300 to activate genes involved in tumor invasiveness, such as Snail and MMP2 [169]. In this case, TRAF6 is critical for TGFβ-induced invasion of cancer cells. Additionally, TRAF6 mediates the suppressive effect of IL-1β or LPS on TGFβ-induced signaling through interaction with the type III TGF-β receptor (TβRIII), an accessory receptor that presents the TGFβ ligand to TβRII. Co-treatment with TGFβ and IL-1β or LPS promotes the interaction between phosphorylated TβRIII and ubiquitinated TRAF6, and thereby sequesters TβRIII from the TβRII/TβRI complex, resulting in inhibition of Smad2/3 activation [170]. Taken together, TRAF6 plays multiple roles in signaling by TGFβ receptors. Interestingly, TGFβ also induces the posttranslational loss of TRAF1, whereas IL-7 restores TRAF1 levels in T cells [171]. No evidence is available about the participation of other TRAFs in TGFβ signaling.

#### IL-2 receptor

The binding of TRAF6 to the TIM of the IL-2R β-chain negatively regulates IL-2-induced Jak1 activation in CD4 T cells, which is likely involved in the proper regulation of T cell activation and development [172].

### TRAFs in other signaling pathways

#### T cell receptor

TRAF1, TRAF3, and TRAF6 are able to regulate signaling by the T cell receptor (TCR). TRAF1 inhibits CD3-induced NF-κB2 activation and proliferation in T cells [31,173]. TRAF3 is recruited to the signaling rafts, and mediates the synergistic activation of ERK, LAT, PLCγ1 and ZAP70 as well as cytokine production and proliferation in T cells following co-stimulation with TCR and CD28 [11]. TRAF6 is also recruited to the TCR signaling rafts containing CARMA1-MALT1-Bcl10-PKCθ-IKK-Caspase 8 via interaction with the paracaspase MALT1, and contributes to the induction of NF-κB activation and IL-2 production in T cells [174,175]. Interestingly, a recent study by Xie *et al* has shown a distinct mechanism of TRAF6 in TCR signaling, in which TRAF6 is recruited to the TCR/CD28 signaling complex by LAT and promotes the ubiquitination and phosphorylation of LAT as well as the activation of NF-AT in T cells [176].

#### C-type lectin receptors

Using macrophages derived from TRAF6^−/−^ mice, it has been shown that TRAF6 is required for NF-κB and JNK activation, and expression of proinflammatory cytokines in response to engagement of C-type lectin receptors during fungal infection [177]. This will elicit further studies of other TRAFs in signaling by C-type lectin receptors.

#### DNA damage response

TRAF6 is essential for DNA damage-induced NF-κB activation. In this process, TRAF6 is activated by the kinase ataxia telangiectasia mutated (ATM), which is a DNA strand break sensor. Following DNA damage, ATM translocates in a calcium-dependent manner to cytosol and membrane fractions, and interacts with TRAF6 via a TIM, resulting in K63-linked polyubiquitination of TRAF6 and recruitment of cIAP1 [178]. The ATM-TRAF6-cIAP1 module stimulates TAB2-dependent TAK1 phosphorylation, and cIAP1 catalyzes monoubiquitination of NEMO at Lys285. NEMO monoubiquitination is a prerequisite for genotoxic NF-κB activation and DNA damage response [178]. Potential involvement of other TRAFs in this response awaits further investigation.

### Substrates, E3 ligases and deubiquitinases of TRAFs

Ubiquitination has emerged as a key regulatory mechanism of TRAFs in signaling. As mentioned above in receptor signaling sections, E3 ligase activity has been demonstrated for TRAF2, TRAF3, TRAF5 and TRAF6, which catalyze non-degradative K63-linked polyubiquitination of their substrates. This is mediated in cooperation with the E2 ubiquitin-conjugating enzymes Ubc13-Uev1A or UbcH5c. It is believed that K63-linked polyubiquitin chains serve as docking sites for formation of signaling complexes, facilitate the recruitment and activation of effector kinases, and thus enable the propagation of signals [9,89,179]. The substrates of TRAFs include TRAF themselves, receptors, kinases, adaptor proteins, transcription factors, E3 ubiquitin ligases, and other functional proteins involved in autophagy or ROS production (Table 2). However, in many cases, substrates of TRAFs (especially those of TRAF2, TRAF3 and TRAF5) have not been unequivocally demonstrated by *in vitro* ubiquitination assays using purified proteins. Interestingly, a recent study has shown that TRAF2 becomes a highly active K63-specific ubiquitin ligase when bound to sphingosine-1-phosphate (S1P), which appears to be a cofactor for TRAF2 E3 ligase activity [180]. This suggests that addition of S1P may improve the efficiency of *in vitro* ubiquitination assays for TRAF2. Future studies need to determine whether similar cofactors exist for TRAF3 and TRAF5. Interestingly, however, the crystal structure of the RING domain of TRAF2 [181] and the phenotype of the ΔRING TRAF2 mutant [61,182] suggest that TRAF2 may not function as an E3 ligase at all. The controversy about whether TRAF2 is actually a RING E3 ligase is described in detail in an excellent review by Silke [183].

**Table 2:** Substrates of the E3 ligase activity of TRAFs

**Substrates (Lys residues of ubiquitination)**	**E3 ligases**	**Receptor signaling**	**References**
**TRAFs**			
TRAF2	TRAF2	TNF-R1/2	[184]
TRAF3	TRAF3	TLR3, TLR4	[92,185]
TRAF5	TRAF5	RIG-I	[139]
TRAF6	TRAF6	TLRs, IL-1R	[17,23,82,89,179,186]
**Receptors**			
IL-17R	TRAF6	IL-17	[156]
p75 (Lys274, 280 and 283)	TRAF6	NGF	[65]
TβRI	TRAF6	TGFβ	[169]
**Kinases**			
TAK1 (Lys158)	TRAF6, TRAF2	TNF-R1/2 and IL-1R	[187]
RIP1 (Lys377)	TRAF2	TNF-R1 and IL-1R	[179,180]
TBK1	TRAF3	TLR3, TLR4	[17,23,81]
IKKε	TRAF3	TLR3, TLR4	[17,23,81]
IRAK1 (Lys134 and 180)	TRAF6	TLR7, TLR9, IL-1R	[179,188,189]
Akt (Lys8 and 14)	TRAF6	IL-1R, IGF-1R	[190]
Fyn (K63)	TRAF6	TLR4	[191]
**Adaptor proteins**			
NEMO (Lys285, 321, 325, 326 and 399)	TRAF6	TLRs, IL-1R, NOD2	[17,23,82,111,192]
TRIF	TRAF2, TRAF6	TLR3, TLR4	[98]
NESCA	TRAF6	TrkA and p75	[193]
LAT (Lys88)	TRAF6	TCR	[176]
**Other E3 ligases**			
cIAP1/2	TRAF2	CD40	[37]
	TRAF6	TLR4-, IL-1R-induced autophagy	[92]
Smurf2	TRAF2	TNF-R2	[194]
**Transcription factors**			
IRF7 (Lys444, 446, and 452)	TRAF6	TLR7, TLR8, TLR9, LMP1, RIG-I	[94,142,195]
IRF5 (Lys410 and 411)	TRAF6	NOD2, TLR7, TLR9	[196,197]
**Regulators of mRNA stability**			
Tristetraprolin	TRAF2	TNF-R1	[198]
**Autophagy proteins**			
Beclin 1 (Lys117)	TRAF6	TLR4-, IL-1R-induced autophagy	[199]
NDP52	TRAF6	TLR3-induced autophagy	[200]
**Regulators of ROS production**			
ECSIT	TRAF6	TLR1, 2, 4-induced ROS production	[93]

While serving as E3 ligases themselves, TRAFs are also substrates of other E3 ligases that catalyze K63-linked or K48-linked polyubiquitination (Table 3). K63-linked polyubiquitination of TRAFs usually leads to protein-protein interactions and promotes signal transduction. For example, Act1-mediated K63-linked ubiquitination of TRAF6 recruits TAB2/3-TAK1 and NEMO to activate NF-κB in IL-17R signaling [201], while cIAP1/2-catalyzed K63-linked ubiquitination of TRAF3 recruits TBK1 and IKKϵ to induce type I IFN production in RIG-I signaling [144]. In an exceptional case, Pellino3-induced ubiquitination of TRAF6 at Lys124 suppresses the ability of TRAF6 to interact with and ubiquitinate IRF7, and thus inhibits type I IFN production in TLR3 signaling [202]. In contrast, K48-linked polyubiquitination of TRAFs results in degradation of TRAF proteins by the 26S proteasome. K48-linked E3 ligases of TRAFs include cIAP1/2, Triad3A, AWP1, SOCS2, Siva-1, Numbl and CHIP. For example, upon viral infection, Triad3A is up-regulated, and induces K48-linked ubiquitination and degradation of TRAF3, thereby forming a negative feedback loop to halt RIG-I signaling and type I IFN production [203]. Thus, K48-linked ubiquitination and subsequent degradation of TRAFs serve as a negative regulatory mechanism of TRAF-dependent signaling.

**Table 3:** E3 ligases that catalyze the ubiquitination of TRAFs

**E3 ligases**	**Target TRAFs (Lys of ubiquitination)**	**Receptor signaling**	**References**
**K-63 linked polyubiquitination**			
Act1	TRAF6 (Lys124)	IL-17R	[201]
	TRAF5	IL-17R	[204]
cIAP1/2	TRAF3 and TRAF6	RIG-I	[144]
Pellino3	TRAF6 (Lys124)	TLR3	[202]
**K-48 linked polyubiquitination**			
cIAP1/2	TRAF2	TNF-R2	[205,206]
	TRAF2	M-CSFR	[207]
	TRAF2 and TRAF3 (Lys107 and Lys156)	CD40 and TLR4	[72,92]
	TRAF3 and TRAF6	RIG-I	[144]
Triad3A	TRAF3	RIG-I	[203]
AWP1	TRAF2	TNF-R1/2	[208]
SOCS2	TRAF6	AhR	[209]
Siva-1	TRAF2	TCR	[210]
Numbl	TRAF6 and TRAF5	IL-1R	[211,212]
CHIP	TRAF2	Cancer cell invasion	[213]

A second negative regulatory mechanism of TRAFs is provided by deubiquitinases that cleave K63-linked polyubiquitin chains from TRAFs, which is just beginning to be understood. The known deubiquitinating enzymes (DUBs) of TRAFs include: (1) ubiquitin-specific proteases, such as CYLD, USP2a, USP4, USP20 and USP25; (2) ovarian tumor (OTU) domain-containing DUBs, such as DUBA (also known as OTUD5), OTUB1, OTUB2, and A20; (3) a novel DUB named monocyte chemotactic protein-induced protein 1 (MCPIP1) (Table 4). CYLD, a tumor suppressor and a target gene of NF-κB, negatively regulates NF-κB and JNK activation by removing K63-linked polyubiquitin chains from TRAF2 and TRAF6 as well as several other signaling proteins [214,215]. Expression of DUBA is up-regulated in TLR and IL-1R stimulated cells. DUBA specifically targets and de-conjugates the K63-linked polyubiquitin chains from TRAF3, resulting in TBK1-IKKϵ dissociation from TRAF3 and inhibition of type I IFN production induced by TLRs and RLRs [128,185,216]. However, DUBA does not affect NF-κB2 activation, which is entirely dependent on K48-linked degradative ubiquitination of TRAF3 [128,185,216]. Interestingly, A20, an unusual enzyme that contains both ubiquitinating and deubiquitinating activities, negatively regulates inflammation by inhibiting NF-κB activation in TNF-R and TLR signaling. A20 is a target gene of NF-κB, and able to remove K63-linked polyubiquitin chains from TRAF6 to turn off NF-κB activation. A20 also inhibits the E3 ligase activities of TRAF6, TRAF2, and cIAP1 by promoting K48-linked polyubiquitination and degradation of the E2 enzymes Ubc13 and UbcH5c [8,128,217]. Furthermore, A20 is capable of targeting an associated signaling molecule such as TRAF2 to the lysosomes for degradation, a process that does not require A20 ubiquitin modifying activity [218]. Notably, A20^−/−^ and MCPIP1^−/−^ mice spontaneously develop severe inflammatory syndrome [219,220], while CYLD^−/−^ and Usp25^−/−^ mice are more susceptible to inflammation [204,221]. Thus, negative regulation of TRAF signaling is necessary to prevent harmful immune responses and inflammatory diseases.

**Table 4:** Deubiquitinating enzymes that target TRAFs

**DUBs**	**TRAFs**	**Receptor signaling**	**References**
**Ubiquitin-specific proteases**			
CYLD	TRAF2, TRAF6	CD40, XEDAR, EDAR, RANK	[222,223]
	TRAF2, TRAF6	IL-1β, TNFα	[224]
USP2a	TRAF2	TNFR1	[225]
	TRAF6	IL-1β, RIG-I	[226]
USP4	TRAF2, TRAF6	TNFα	[227]
	TRAF6	TLR4, IL-1R	[228]
USP20	TRAF6	IL-1β	[229]
USP25	TRAF5, TRAF6	IL-17R	[204]
**Ovarian tumor (OTU) family of DUBs**			
DUBA (OTUD5)	TRAF3	IL-1β, TLR9	[216]
	TRAF3	TLR3, TLR4, TLR7, RIG-I, MDA-5	[185]
OTUB1	TRAF3, TRAF6	RIG-I	[230]
OTUB2	TRAF3, TRAF6	RIG-I	[230]
A20	TRAF6	TLR4, TLR2	[231,232]
**Novel cellular DUBs**			
MCPIP1	TRAF2, TRAF3, TRAF6	IL-1, TLR4	[219]

In addition to ubiquitination, other post-translational modifications, including phosphorylation and glutathionylation, are also reported to regulate TRAFs in signaling. Phosphorylation of TRAF1 (at Ser 139 in mouse and Ser 146 in human by PKN1) inhibits TNF-R2-dependent tonic NF-κB and JNK signaling in HeLa cells [233], and also has a negative impact on the recruitment of TBK1 to the 4-1BB signaling complex and the subsequent NF-κB activation in T cells [234]. Phosphorylation of TRAF2 (at Ser11 and Ser55 by PKCζ or IKKϵ, and at Thr117 by PKCδ and PKCϵ), which promotes K63-linked ubiquitination of TRAF2 and NF-κB activation, has been demonstrated in TNFα signaling or in transformed cells [235-238]. Following NOD2 activation, phosphorylation of TRAF4 (at Ser 426 by IKKα) negatively regulates NOD2 signaling in macrophages, including NF-κB activation, cytokine production and antibacterial activity [113]. Tyrosine phosphorylation of TRAF6 by Fyn and c-Src has been shown following LPS stimulation [191]. Interestingly, a recent study reported that TRAF6 is S-glutathionylated under normal conditions. Upon IL-1 stimulation, TRAF6 undergoes deglutathionylation catalyzed by glutaredoxin-1 (GRX-1), a process that is essential for TRAF6 auto-ubiquitination and subsequent NF-κB activation [239]. These findings suggest that different post-translational modifications of TRAF proteins coordinate to regulate the activity of TRAFs in signaling in a dynamic manner.

### Viral proteins that target or hijack TRAFs

TRAFs are critical players in host immunity, as demonstrated by their shared usage by both innate immune receptors (such as TLRs, NLRs, RLRs, and cytokine receptors) and adaptive immune receptors (such as CD40, BAFF-R, OX40, 4-1BB, and TCR). Interestingly, viruses and bacteria have developed a variety of strategies to target or hijack TRAFs to evade host immune responses and to promote their own propagation or persistence (Table 5). (1) Several viral and bacterial proteins can function as DUBs to deubiquitinate TRAFs and thus inhibit type I IFN production in RIG-I or TLR signaling. Examples include Lb(pro) of foot-and-mouth disease virus, X protein (HBx) of hepatitis B virus, and YopJ of the Gram- bacterium Yersinia pestis [21,90,240,241]. (2) Some viral proteins can specifically interact with TRAFs and disrupt the formation of TRAF signaling complexes. For example, Gn protein of NY-1 hantavirus and M protein of severe acute respiratory syndrome (SARS) coronavirus disrupt or prevent the formation of TRAF3-TBK1-IKKϵ complex to inhibit type I IFN production [242,243]. Similarly, A52R and K7 proteins of vaccinia virus disrupt signaling complexes containing TRAF6 and IRAK2 to block NF-κB activation and antiviral defense [20,244]. (3) Some viral proteins usurp TRAFs for viral signaling to promote their own propagation or persistence. The best example of this group is latent membrane protein 1 (LMP1) of Epstein-Barr virus, which sequesters most cellular TRAF3, and hijacks TRAF1, 2, 3, 5 and 6 to mimic constitutively activated CD40 signaling [245-249]. (4) The v-FLIP member MC159 of the human molluscum contagiosum virus mediates the recruitment of both TRAF2 and TRAF3 into the Fas death inducing signaling complex to modulate Fas signaling, and powerfully inhibits both caspase-dependent and caspase-independent cell death induced by Fas [250]. (5) Some viruses up-regulate the expression of specific miRNAs to target TRAFs. For example, the Tat protein of HIV-1 and VSV infection up-regulate miR-32 and miR-146a, which directly target the protein expression of TRAF3 and TRAF6, respectively [251-253]. Together, the above evidence further highlights the crucial importance of TRAFs in host immunity against pathogens.

**Table 5:** Pathogenic proteins that target TRAFs

**Viral or bacterial proteins**	**TRAFs**	**Mechanisms**	**Ref.**
**Function as DUBs of TRAFs**			
Lb(pro) of foot-and-mouth disease virus	TRAF3, TRAF6	Deubiquitinates TRAF3 and 6 to inhibit RIG-I signaling	[21]
X protein (HBx) of hepatitis B virus	TRAF3	Deubiquitinates TRAF3 to inhibit RIG-I signaling	[240]
YopJ of the Gram- bacterium Yersinia pestis	TRAF3, TRAF6	Deubiquitinates TRAF3 and 6 to inhibit TLR signaling	[241]
**Disrupt the formation of TRAF signaling complex**			
Gn protein of NY-1 hantavirus	TRAF3	Disrupts the interaction of TRAF3 and TBK1-IKKε	[242]
M protein of severe acute respiratory syndrome (SARS) coronavirus	TRAF3	Prevents the formation of TRAF3-TBK1-IKKε complex	[243]
A52R of vaccinia virus	TRAF6	Disrupts the signaling complex of TRAF6 and IRAK2	[244]
K7 of vaccinia virus	TRAF6	Disrupts the signaling complex of TRAF6 and IRAK2	[20]
**Usurp TRAFs for viral signaling**			
LMP1 of Epstein-Barr virus	TRAF1, 2, 3, 5, 6	Sequesters cellular TRAF3, and usurps TRAF1, 2, 3, 5	[245-249]
		and 6 to mimic constitutively activated CD40 signaling	
BRRF1 lytic gene product (Na) of Epstein-Barr virus	TRAF2	Utilizes TRAF2 for JNK activation and lytic gene	[254]
		expression	
v-FLIP of Kaposi’s sarcoma herpesvirus (human herpesvirus 8)	TRAF2, TRAF3	Recruits TRAF2 and 3 to activate NF-κB and JNK, and	[255]
		to induce cell survival in primary effusion lymphomas	
U(L)37 tegument protein of the herpes simplex virus (HSV)	TRAF6	Activates TRAF6 and NF-κB to induce IL-8 expression	[256]
**Modify TRAF signaling complex**			
MC159 of human molluscum contagiosum virus	TRAF2, TRAF3	Recruits TRAF2 and 3 to Fas signaling complex	[250]
		and inhibits Fas-induced apoptosis	
**Induce miRNAs to target TRAFs**			
Tat protein of HIV-1	TRAF3	Up-regulates miR-32 that directly targets TRAF3	[251]
VSV	TRAF6	Up-regulates miR-146a that targets TRAF6 and IRAK1	[252]

### *In vivo* functions of TRAFs in mice

The *in vivo* functions of TRAFs in whole animals have been explored by gene targeting in mice. Mice genetically deficient in individual TRAFs have been generated. Among these knockout mice, only TRAF1^−/−^, TRAF5^−/−^, and 67% of TRAF4^−/−^ mice could survive to adulthood. In contrast, mice deficient in TRAF2, 3, or 6 exhibit perinatal death with multiple organ abnormalities, indicating that TRAF2, 3, and 6 are indispensable in early development. Although viable, mice deficient in TRAF1, 4 or 5 exhibit distinct phenotypes (Table 6). For example, skin of TRAF1^−/−^ mice is hypersensitive to TNF-induced necrosis [173], and these mice are resistant to allergic lung inflammation in an experimental model of asthma [257]. TRAF4^−/−^ mice suffer respiratory disorder and wheezing caused by tracheal ring disruption, and exhibit numerous developmental abnormalities, including defects in the development of the axial skeleton and in the closure of the neural tube as well as myelin perturbation [258-260]. For mice with early lethality, the causes of death appear to be different. TRAF2^−/−^ mice succumb to severe colitis that result from apoptosis of colonic epithelial cells and accumulation of IL-10-secreting neutrophils, which can be ameliorated by deletion of TNFR1 or combined treatment with neutralizing antibodies against TNFα and IL-10 [261,262]. The early lethality of TRAF3^−/−^ mice is rescued by compound loss of the NF-κB2 gene, suggesting that constitutive NF-κB2 activation leads to the lethal phenotype of TRAF3^−/−^ mice [263]. In contrast, TRAF6^−/−^ mice die of severe osteopetrosis, splenomegaly, and thymic atrophy [264,265]. Taken together, these findings demonstrate that although TRAFs have overlapping functions, each TRAF molecule also plays unique and distinct roles that could not be compensated or substituted by other TRAFs in whole animals.

**Table 6:** In vivo functions of TRAFs in mice

									**Genotype**					**Type of knockout**																																													**Phenotype**	**References**
									**TRAF1**					
									TRAF1−/−					Germline																																													Viable and normal lymphocyte development	[173]
																																																											Skin hypersensitive to TNF-induced necrosis	[173]
																																																											Hyperproliferation in response to T cell receptor signaling	[173]
																																																											Enhanced Th2 responses	[266]
																																																											Lack of 4-1BB-mediated survival responses in CD8 and memory T cells	[78,79,171]
																																																											Required for 4-1BB-induced NF-κB1 activation in T cells	[31]
																																																											Constitutive NF-κB2 activation in CD8 T cells	[31]
									**TRAF2**																																																			
									TRAF2−/−					Germline																																													Progressively runted and die within 3 weeks after birth	[267]
																																																											Atrophy of the thymus and spleen; depletion of B cell precursors	[267]
																																																											Elevated serum TNF levels; cells sensitive to TNF-induced apoptosis	[267]
																																																											Severe reduction in TNF-mediated JNK activation	[267]
																																																											Severe colitis; drastic changes in the colonic microbiota	[261]
																																																											Increased number of Th17 cells in the colonic lamina propria	[261]
																																																											Apoptosis of colonic epithelial cells due to TNFR1 signaling	[261]
																																																											IL-10-secreting neutrophils accumulated in peripheral blood and bone marrow	[262]
									TRAF2flox/flox, CD19-Cre					B cell-specific																																													Prolonged B cell survival independent of BAFF	[36]
																																																											Splenomegaly and lymphadenopathy	[36]
																																																											Constitutive NF-κB2 activation in B cells	[36]
																																																											Slower and decreased CD40-induced phosphorylation of JNK, p38 and ERK	[74]
																																																											Reduced germinal center formation following SRBC immunization	[74]
									TRAF2flox/flox, Lck-Cre					T cell-specific																																													Normal T cell survival; constitutive NF-κB2 activation in T cells	[36]
									TRAF2flox/flox, Albumin-Cre					Hepatocyte-specific																																													Severely impaired hyperglycemic response to glucagon	[268]
									**TRAF3**																																																			
									TRAF3−/−					Germline																																													Progressively runted; die by 10 days after birth	[269]
																																																											Impaired T cell responses	[269]
									TRAF3flox/flox, CD19-Cre					B cell-specific																																													Prolonged B cell survival independent of BAFF	[36,270]
																																																											Splenomegaly and lymphadenopathy	[36,270]
																																																											Autoimmune manifestations and hyperimmunoglobulinemia	[270]
																																																											Increased T-independent antibody responses	[270]
																																																											Development of B1 lymphomas and splenic marginal zone lymphomas	[271]
																																																											Enhanced signaling by TLR3, TLR4, TLR7, and TLR9 in B cells	[272]
																																																											Accelerated CD40-induced phosphorylation of JNK, p38 and ERK	[74]
									TRAF3flox/flox, Lck-Cre					T cell-specific																																													Normal T cell survival; constitutive NF-kB2 activation in T cells	[36]
									TRAF3flox/flox, CD4-Cre					T cell-specific																																													Normal T cell survival; constitutive NF-kB2 activation in T cells	[11]
																																																											Normal CD4 and CD8 T cell development; increased number of Treg cells	[11]
																																																											Defective T-dependent antibody responses	[11]
																																																											Impaired T cell-mediated immunity to bacterial infection	[11]
																																																											Defective T cell responses to co-stimulation by T cell receptor and CD28	[11]
									**TRAF4**																																																			
									TRAF4−/−					Germline																																													Embryonic lethal but with great individual variation	[258,259]
																																																											Respiratory disorder and wheezing caused by tracheal ring disruption	[258,259]
																																																											Surviving mutant mice manifest numerous developmental abnormalities	[258,259]
																																																											Altered locomotion coordination typical of ataxia	[258,259]
																																																											High incidence of spina bifida	[258,259]
																																																											Degeneration of a high number of Purkinje cells	[260]
																																																											Increased rates of pulmonary inflammation	[260]
																																																											Reduced migration of DCs; normal development of T and B lymphocytes	[273]
																																																											Inhibits IL-17 signaling and Th17-mediated autoimmune encephalomyelitis	[164]
									**TRAF5**																																																			
									TRAF5−/−					Germline																																													Viable and normal development	[274]
																																																											Mild defect in T-dependent antibody responses	[274]
																																																											Defective in Th1/Th2 differentiation	[275]
									**TRAF6**																																																			
									TRAF6−/−					Germline																																													Perinatal and postnatal lethality	[264,265]
																																																											Severe osteopetrosis; defective in osteoclast formation	[264,265]
																																																											Defective IL-1, CD40 and LPS signaling in lymphocytes	[264,265]
																																																											Defective in lymph node organogenesis	[265]
																																																											Reduced number of immature B cells in the bone marrow	[265]
																																																											Severe defect in the Treg development in thymus	[276]
																																																											Defective development, maturation and activation of DCs	[277]
																																																											Impaired cytokine production in mast cells following FcεRI aggregation	[278]
																																																											Hypohidrotic ectodermal dysplasia	[279]
									TRAF6flox/flox, CD19-Cre					B cell-specific																																													Reduced number of mature B cells in the bone marrow and spleen	[280]
																																																											Impaired T-dependent and T-independent antibody responses	[280]
																																																											Lack of CD5+ B-1 cells	[280]
									TRAF6flox/flox, CD4-Cre					T cell-specific																																													Multiorgan inflammatory disease; hyperactivation of the PI3K-Akt pathway	[281]
																																																											Resistant to suppression by CD4+CD25+ regulatory T cells	[281]
																																																											Resistant to anergizing signals	[282]
																																																											A profound defect in generating CD8 memory T cells;	[283]
																																																											Defective AMPK activation and mitochondrial fatty acid oxidation	[283]
																																																											Specific increase in Th17 differentiation	[284]
																																																											More sensitive to TGFβ-induced Smad2/3 activation and proliferation arrest	[284]
																																																											A severe defect in the Treg development	[276]
									TRAF6flox/flox, MCK-Cre					Skeletal muscle-specific																																													Improved muscle preservation in response to starvation or cancer cachexia	[60,285,286]
																																																											Improved regeneration of myofibers upon injury	[60,285,286]
																																																											Augmented the M2 macrophage phenotype in injured muscle tissues	[60,285,286]
																																																											Upregulated Notch signaling and reduced inflammatory cytokine production	[287,288]

During the past few years, different laboratories have employed the conditional gene targeting strategy to circumvent the early lethality of TRAF^−/−^ mice. These new mouse models allow more detailed analyses and direct comparison of specific functions of TRAFs in different cell types of whole animals (Table 6).

#### B lymphocytes

TRAF2, 3, 5 and 6 are important in the survival, development, and activation of B cells. In the absence of either TRAF2 or TRAF3, B cells exhibit remarkably prolonged survival independent of BAFF, which result from the constitutive NF-κB2 activation [36,270,271]. This is further corroborated by the evidence that deletion of cIAP1 and cIAP2 (constitutive interacting partners of TRAF2) also renders BAFF-independent survival of B cells in mice due to constitutive NF-κB2 activation [74]. Strikingly, the development of mature B cells, including the follicular and marginal zone subpopulations of the spleen, are unimpaired in BAFF-R^−/−^ mice that also lack B cell expression of either TRAF2, TRAF3, or cIAP1/cIAP2 [74]. Thus, the survival and maturation pathways normally activated by physiologic triggering of BAFF-R by BAFF are constitutively activated when the expression of TRAF2, TRAF3, or cIAP1/cIAP2 is absent from B cells [74]. Vastly prolonged survival of B cells eventually leads to autoimmune manifestations and B lymphoma development in B cell-specific TRAF3^−/−^ mice [270,271]. Interestingly, TRAF3^−/−^ B cells also display enhanced activation in response to signaling by TLR3, TLR4, TLR7, or TLR9 [272]. Gardam *et al*. further directly compared CD40 signaling in B cell-specific TRAF3^−/−^, TRAF2^−/−^, and cIAP1^−/−^cIAP2^−/−^ mice [74]. Interestingly, loss of TRAF2, TRAF3, or cIAP1/cIAP2 in B cells has very different impacts on CD40 signaling. TRAF3^−/−^ B cells exhibit accelerated phosphorylation of JNK, ERK, and p38 in response to CD40 signaling. In contrast, TRAF2^−/−^ B cells display slower and decreased CD40 signaling, while cIAP1^−/−^cIAP2^−/−^ B cells show impaired CD40 signaling [74]. Consistent with this, B cell-specific TRAF2^−/−^ and cIAP1^−/−^cIAP2^−/−^ but not TRAF3^−/−^ mice exhibit dramatically reduced germinal center formation following immunization with sheep red blood cells [74]. Notably, TRAF5^−/−^ B cells show defects in proliferation and up-regulation of surface molecules in response to CD40 stimulation, and reduced production of IgM and IgG1 in response to stimulation with CD40 plus IL-4 [274]. Unexpectedly, TRAF6 ablation results in defects in generation of CD5+ B1 cells, reduced number of mature B cells in the bone marrow and spleen, and impaired T-dependent and T-independent antibody responses [280].

#### T lymphocytes

TRAFs (except TRAF4) play critical roles in regulating T cell immunity. TRAF1^−/−^ T cells exhibit hyperproliferation and increased production of Th2 cytokines (IL-4, IL-5 and IL-13) in response to TCR signaling, but defective 4-1BB-mediated survival responses in effector and memory CD8 T cells [77-79,171,266]. Hyperproliferation of TRAF1^−/−^ T cells is due to constitutive activation of the NF-κB2 pathway [31]. Paradoxically, TRAF2^−/−^ or TRAF3^−/−^ T cells display neither prolonged survival (as that observed in B cells) nor hyperproliferation (as that observed in TRAF1^−/−^ T cells), despite their constitutive processing and activation of NF-κB2 [11,36]. However, the TRAF2-NIK-NFκB2 pathway does drive the development of fatal autoimmune inflammatory disorder in TRAF2^−/−^TNFα^−/−^ mice [289]. Surprisingly, T cell-specific TRAF3^−/−^ mice have increased frequency of regulatory T (Treg) cells, and exhibit defective T-dependent IgG1 responses and T cell-mediated immunity to infection with *Listeria monocytogenes*, which is due to impaired TCR/CD28 signaling [11]. Similarly, CD27-mediated co-stimulatory signaling was reduced in TRAF5^−/−^ T cells [274]. In contrast, TRAF6^−/−^ mice show a severe defect in Treg development in thymus [276]. T cell-specific deletion of TRAF6 results in the development of multiorgan inflammatory disease [281]. TRAF6^−/−^ T cells exhibit hyperactivation of the PI3K-Akt pathway, resistance to suppression by Treg cells, and also resistance to anergizing signals [281,282]. TRAF6^−/−^ CD4 T cells display increased Th17 differentiation, due to enhanced sensitivity to TGFβ-induced Smad2/3 activation and IL-2 down-regulation [284]. Interestingly, activated TRAF6^−/−^ CD8 T cells exhibit defective AMP-activated kinase activation and mitochondrial fatty acid oxidation (FAO) in response to growth factor withdrawal, resulting in a profound defect in memory CD8 T cell development after infection [283].

#### DCs and mast cells

TRAF1, 2, 3, 4 and 6 regulate the functions of dendritic cells (DCs). Arron *et al*. demonstrated the cooperation of TRAF1 and TRAF2 in DCs [290]. TRAF1^-/-^ DCs matured in CD154 display impaired NF-κB activation and survival but increased TRAF2 degradation in response to CD154 re-stimulation [290]. TRAF3^−/−^ DCs produce increased amounts of IL-12 but reduced amounts of IL-10 and little type I IFN in response to TLR7 and TLR9 signaling [18,95,96]. TRAF3^−/−^ DCs also display constitutive NF-κB2 activation but not prolonged survival [18]. TRAF4^−/−^ DCs exhibit reduced migration both in transwell experiments and *in vivo*[273]. Interestingly, TRAF6 is required for DC maturation and activation. In response to either microbial components or CD40L, TRAF6^−/−^ DCs fail to up-regulate surface expression of MHC class II and CD86, or produce inflammatory cytokines [277]. Similarly, TRAF6^−/−^ mast cells exhibit impaired production of IL-6, CCL-9, IL-13, and TNF following FcϵRI aggregation by IgE [278].

#### Hepatocytes and skeletal muscles

Hepatocyte-specific TRAF2^−/−^ mice exhibit significantly decreased blood glucose levels under high-fat diet conditions. Although these mice show normal insulin signaling and the hypoglycemic response to insulin, they have severely impaired glucagon signaling and the hyperglycemic response to glucagon. In addition, TRAF2 overexpression significantly increases the ability of glucagon or a cAMP analog to stimulate CREB phosphorylation, gluconeogenic gene expression, and hepatic glucose production in primary hepatocytes. Thus, hepatic cell TRAF2 autonomously promotes hepatic gluconeogenesis, and contributes to hyperglycemia in obesity [268]. Interestingly, skeletal muscle-specific depletion of TRAF6 in mice improves satellite cell activation and skeletal muscle regeneration through up-regulation of Notch signaling and reducing the inflammatory repertoire [287]. TRAF6 deficiency inhibits the induction of atrophy program in response to starvation, denervation, or cancer cachexia by suppressing the expression of key regulators of atrophy, including MAFBx, MuRF1, p62, LC3B, Beclin1, Atg12, and Fn14 [60,285,286]. Ablation of TRAF6 also improves the phosphorylation of Akt and FoxO3a and inhibits the activation of 5′ AMP-activated protein kinase in skeletal muscle in response to starvation. Moreover, K63-linked autoubiquitination of TRAF6 regulates ER stress and unfolding protein response pathways in starvation-induced muscle atrophy [288]. It remains to be elucidated whether other TRAFs regulate hepatocyte and skeletal muscle functions.

#### Atherosclerosis

Experiments of mouse models of atherosclerosis have provided evidence that TRAF1, 5 and 6 regulate the pathogenesis of this disease. Missiou *et al*. reported that TRAF1 deficiency attenuates atherosclerosis in low-density lipoprotein receptor (LDLR)^−/−^ mice by impairing monocyte recruitment to the vessel wall [291]. Deletion of TRAF1 inhibits adhesion of inflammatory cells to the endothelium, reduces the expression of CD29 in macrophages, and decreases the expression of the adhesion molecules ICAM-1 and VCAM-1 in endothelial cells [291]. In contrast, TRAF5 deficiency accelerates atherogenesis in LDLR^−/−^ mice. Deletion of TRAF5 in endothelial cells or in leukocytes enhances adhesion of inflammatory cells to the endothelium, thus facilitating inflammatory cell recruitment to the atherosclerotic plaques. In addition, TRAF5 deficiency increases the expression of adhesion molecules and chemokines, and potentiates macrophage lipid uptake and foam cell formation [292]. Interestingly, endothelial and myeloid cell TRAF6 proteins have opposite roles in atherosclerosis in ApoE^−/−^ mice. Endothelial TRAF6 deficiency inhibits atherosclerosis by reducing proinflammatory gene expression and monocyte adhesion to endothelial cells. In contrast, myeloid cell-specific TRAF6 deletion exacerbates atherosclerosis. TRAF6^−/−^ macrophages exhibit impaired expression of the atheroprotective cytokine IL-10, elevated ER stress, increased sensitivity to oxidized LDL-induced apoptosis, and reduced capacity to clear apoptotic cells [293]. Similar mouse models of TRAF2, 3 and 4 need to be generated and characterized in future studies.

### TRAFs in human diseases

Findings obtained from TRAF-deficient mouse models have laid the basis to understand the roles of TRAFs in the pathogenesis of human diseases. Given their importance in regulating the development, survival and activation of various cell types, it would be expected that aberrant functions of TRAFs may contribute to different diseases. However, the roles of TRAFs in human diseases are just beginning to be revealed. Available evidence implicates TRAFs in the pathogenesis of cancers, autoimmune diseases, immunodeficiencies, and neurodegenerative diseases (Table 7).

**Table 7:** Genetic variations of TRAFs in human diseases

**Diseases**	**Genetic variations of TRAFs**	**References**
**B cell malignancies**		
Multiple myeloma	Deletions or inactivating mutations of TRAF3, TRAF2	[294-296]
	SNPs of TRAF3	[297]
Splenic marginal zone lymphoma	Deletions or inactivating mutations of TRAF3	[298,299]
B cell chronic lymphocytic leukemia	Deletions or inactivating mutations of TRAF3	[298]
Mantle cell lymphoma	Deletions or inactivating mutations of TRAF3	[298]
Waldenström’s macroglobulinemia	Deletions or inactivating mutations of TRAF3	[300]
Hodgkin lymphoma	Deletion of TRAF3	[301]
Diffuse large B-cell lymphoma	Inactivating mutations of TRAF2, TRAF5	[302]
Non-Hodgkin lymphoma	SNPs of TRAF1	[303]
**Carcinomas**		
Breast cancers	Amplification of TRAF4	[304]
Lung cancers	Amplification of TRAF4, TRAF6	[304,305]
Osteosarcoma	Amplification of TRAF6	[306]
**Autoimmune diseases**		
Systemic lupus erythematosus	SNPs of TRAF6, TRAF1/C5	[22,307,308]
Rheumatoid arthritis	SNPs of TRAF5, TRAF6, TRAF1/C5	[22,309-311]
**Immunodeficiencies**		
HSV-1 encephalitis	Inactivating mutation of TRAF3	[312]
**Other**		
Hypohidrotic ectodermal dysplasia	Inactivating mutation of TRAF6	[313]

#### B cell malignancies

Growing literature documents the prominent relevance of TRAF3, TRAF2 and TRAF1 in B cell malignancies. As predicted from their critical roles in inhibiting B cell survival, biallelic deletions or inactivating mutations of TRAF3 and TRAF2 frequently occur in primary human samples of B cell neoplasms. Deletions and mutations of TRAF3 have been reported in multiple myeloma [294-296], Waldenström’s macroglobulinemia [300], Hodgkin lymphomas (HLs) [301], and a variety of non-Hodgkin lymphomas (NHLs), including splenic marginal zone lymphoma, B cell chronic lymphocytic leukemia (B-CLL), and mantle cell lymphoma [298,299]. Similarly, inactivating mutations of TRAF2 have been identified in multiple myeloma [294-296] and diffuse large B-cell lymphoma (DLBCL) [302]. Single nucleotide polymorphisms (SNPs) of TRAF3 are also associated with altered risk of multiple myeloma [297]. In contrast, TRAF1 expression is ubiquitously elevated in both HLs [314] and NHLs, especially in CLL and mediastinal large B-cell lymphoma [315-317]. In addition, TRAF1 SNPs are associated with NHLs [303]. Thus, TRAF3 and TRAF2 are tumor suppressive, whereas TRAF1 appears to be oncogenic in B cells.

#### Carcinomas

Overexpression and gene amplification of TRAF4 and TRAF6 have been reported in human carcinomas. TRAF4 is overexpressed in breast and lung carcinomas [304,318,319]. TRAF4 protein overexpression is limited to cancer cells and the subcellular localization is consistently cytoplasmic in a large majority of cases. Increased TRAF4 gene copy number is one major mechanism responsible for TRAF4 protein overexpression in human cancers. Indeed, TRAF4 is located at chromosome 17q11.2 in a region of amplification devoid of other known oncogenes [304,318,319]. Intriguingly, TRAF4 is a target gene of the p53 family of transcription factors, including p63, p73 and p53, in squamous cell carcinoma of the head and neck (SCCHN). TRAF4 locates in the nucleus in normal oral epithelium and highly/moderately differentiated cells, but is localized in the cytoplasm in poorly differentiated SCCHN. Overexpression of TRAF4 in SCCHN induces apoptosis and suppresses colony formation [320-322]. Thus, TRAF4 overexpression has different outcomes in different carcinomas. Notably, TRAF6 gene is located in another frequently amplified region at chromosome 11p13. TRAF6 exhibits overexpression and gene amplification in lung cancer and osteosarcoma cells [305,306,323]. Downregulation of TRAF6 in human lung cancer and osteosarcoma cells suppresses NF-κB activation, cell survival and proliferation, and tumor formation and invasion. These observations suggest that TRAF6 overexpression may promote the tumorigenesis and invasion of lung cancer and osteosarcoma cells [305,306,323].

#### Autoimmune diseases

Single nucleotide polymorphisms (SNPs) in TRAFs have been linked to autoimmune diseases such as systemic lupus erythematosus (SLE) and rheumatoid arthritis (RA). SNPs of TRAF6 are associated with both SLE and RA [22]. Similarly, SNPs at the TRAF1/C5 locus are associated with both SLE and RA [307,308,310,311]. A single SNP (rs7514863), mapping upstream of the TRAF5 gene and affecting a putative transcription factor binding site, demonstrates a significant association with RA [309]. In addition, decreased expression of TRAF2 has been detected in peripheral blood mononuclear cells of SLE patients [324]. However, further association and functional studies are required to determine whether these TRAFs play causal roles in increasing susceptibility to SLE or RA.

#### Immunodeficiencies

An autosomal dominant mutation of TRAF3 has been reported in a young adult with a history of herpes simplex virus-1 (HSV-1) encephalitis in childhood [312]. The TRAF3 mutant allele is loss-of-expression, loss-of-function, dominant-negative, and associated with impaired responses upon stimulation of both TNF-Rs and TLRs. The recurrent HSV-1 infection and encephalitis result from the impairment of TLR3-induced type I IFN production [312].

#### Hypohidrotic ectodermal dysplasia

A heterozygous mutation of TRAF6 has recently been identified in a patient with hypohidrotic ectodermal dysplasia (HED)[325]. The mutant TRAF6 protein is capable of forming a complex with TAK1 and TAB2, but cannot bind to the receptor XEDAR. Furthermore, the mutant TRAF6 protein potently inhibits the interaction between wild type TRAF6 and XEDAR, and suppresses the XEDAR-mediated NF-κB activation. Thus, this mutant TRAF6 protein acts in a dominant-negative manner to affect the XEDAR-mediated NF-κB activation during the development of ectoderm-derived organs, leading to HED phenotype [313].

#### Neurodegenerative diseases

Interestingly, recent evidence implicates the E3 ligase activity of TRAF6 in the pathogenic aggregation of mutant proteins in neurodegenerative diseases such as Parkinson’s disease and Huntington disease. It was found that TRAF6 binds to misfolded mutant DJ-1, aSYN and N-HTT, proteins involved in the pathogenesis of the Parkinson’s disease and Huntington disease. Mutant DJ-1, aSYN and N-HTT proteins are all substrates of TRAF6. Instead of conventional K63-linked polyubiquitination, TRAF6 promotes atypical ubiquitination of DJ-1, aSYN and N-HTT with K6, K27, and K29 linkage formation, thereby stimulating aggregate formation of mutant DJ-1, aSYN and N-HTT in neurodegenerative diseases [326,327].

#### Chronic inflammation and infection

In light of their crucial importance in inflammatory and immune responses, it would be predicted that TRAF molecules may also contribute to chronic inflammation and infection. Although no genetic association of TRAFs and chronic inflammation or infection has been identified, recent evidence of alterations of TRAF protein levels supports this possibility. Notably, TRAF2 and TRAF3 are often degraded in response to signaling by the TNF-R superfamily [3,27,32,328]. In contrast, TRAF1 expression is up-regulated by NF-κB activation in response to signaling by a variety of receptors, including TNF-R superfamily and cytokine receptors, etc. [329-331]. The dynamic change of the stoichiometry of different TRAF molecules inside the cell impacts subsequent cellular responses to inflammatory or infectious stimuli. For example, the presence of TRAF1 stabilizes TRAF2, which plays a role in promoting proinflammtory responses in HeLa cells [332,333]. More direct evidence was provided by a recent study demonstrating that TRAF1 is specifically lost from virus-specific CD8 T cells during the chronic phase of infection with HIV in humans [171]. This area warrants further investigation.

## Conclusions

Since the first TRAFs were cloned in the mid 1990s, we have witnessed a remarkable progress in understanding the functions of TRAFs in signaling. TRAFs are now recognized as signal transducers of a wide variety of receptors, including the TNF-R superfamily, TLRs, NLRs, RLRs, IL-1R family, IL-17Rs, IFN receptors, TGFβ receptors, IL-2R, TCR, and C-type lectin receptors. Although initially defined as adaptor proteins, most TRAFs also function as E3 ubiquitin ligases through their RING finger domain. Furthermore, activation of TRAFs is exquisitely regulated by post-translational modifications, especially ubiquitination, which has become the subject of intense investigations during the past few years. Termination of TRAF activation could be achieved through either K48-linked polyubiquitination followed by proteosomal degradation or removal of K63-linked polyubiquitin chains catalyzed by deubiquitinases. Acting alone or in combination, TRAF-dependent signaling pathways regulate the activation of NF-κBs, MAPKs, or IRFs to control diverse cellular processes. Accumulating evidence obtained from TRAF-deficient mice demonstrates that each TRAF plays obligatory and distinct roles critical for innate immunity, adaptive immunity, embryonic development, and tissue homeostasis. The pivotal roles of TRAFs in host immunity are further highlighted by the finding that targeting TRAFs appears to be a common mechanism employed by pathogenic proteins of viruses and bacteria. Furthermore, the interest in TRAFs is also driven by recent discoveries that link TRAF genetic variations to human diseases such as cancers, autoimmune diseases, and immunodeficiencies. In conclusion, TRAFs are versatile and indispensable regulators of signal transduction and immune responses, and aberrant functions of TRAFs contribute to the pathogenesis of human diseases.

### Perspectives

Despite the wealth of current knowledge about TRAFs, many key questions remain, which will drive the next stage of research in this important area. (1) What is the stoichiometric composition of TRAFs and other signaling proteins in each signaling complex? What are the dynamic kinetics of activation and spatial regulation of each TRAF molecule in response to each specific stimulus? Cutting-edge biochemical, proteomic, and imaging technologies will be needed to uncover these details. (2) How is the E3 ligase activity of each TRAF regulated precisely? What are the substrates of the E3 ligase activities of TRAF2, 3 and 5? Are there additional E3 ligases, deubiquitinases, kinases, and phosphatases that target different TRAFs? Are the enzymes targeting TRAFs regulated by TRAF-dependent signaling pathways? *In vitro* reconstitution experiments and ligase activity assays, high throughput screens for substrates and enzymes, and systems biology approaches will be needed to address these issues. (3) What are the molecular structures of each TRAF in complex with its specific signaling partner, substrate, or enzyme? This requires access to co-crystals containing TRAFs and their interacting partners, and the crystal structure of full-length TRAFs/substrates remains a challenge. (4) Are there additional pathogenic factors of invading microorganisms that target TRAFs during infections? If so, by what precise mechanisms? Yeast 2-hybrid screen, bioinformatic studies and proteomic approaches may be applied toward this end. (5) During pathogen infections, multiple TRAF-dependent signaling pathways are triggered either sequentially or simultaneously, including innate immune receptors (such as TLRs, NLRs and RLRs), adaptive immune receptors (such as TCR, CD40, OX-40 and 4-1BB), and cytokine receptors (such as IL-1R, IL-17R, IFN-Rs, and TβRs). How does each TRAF act in such complex and concerted signaling pathways in different cellular context during infection? Whether and how does each TRAF regulate the crosstalk between different immune signaling pathways? Responses of TRAF^−/−^ mice, and especially cell type-specific TRAF^−/−^ mice, to infections will be instrumental in addressing these questions. Sequential or simultaneous co-engagement of different immune receptors also needs to be investigated thoroughly in cultured cells. (6) What are the cell type-specific factors that dictate cell type-specific TRAF functions? For example, TRAF2 or TRAF3 deficiency leads to prolonged survival in B cells, but not in T cells or DCs. Genetic and systems biology approaches will be required for such studies. (7) Are there additional TRAF genetic alterations and SNPs associated with human diseases? Do epigenetic modifications of TRAFs contribute to disease conditions? How? Systematic and comprehensive analyses employing genetic, bioinformatic, and deep sequencing approaches will facilitate such investigation. Generation and examination of TRAF^−/−^ or TRAF-transgenic mouse models of human diseases are also required to decipher the underlying mechanisms. Together, these future studies will undoubtedly yield valuable information to advance our understanding of TRAFs.

Given the importance of TRAFs in host immunity and in human diseases, the above future studies will also provide a platform for the development of therapeutic intervention of TRAF-mediated human diseases. For example, insights gained into the structures of each TRAF in complex with its specific signaling partner, substrate, or enzyme will guide the development of structure-based therapeutics. Small agonists and antagonists of TRAFs may be devised to enhance beneficial signaling pathways and to interfere with harmful ones, respectively. In this regard, cell-permeable TRAF6 decoy peptides potently inhibit TRAF6 signaling in cultured cells, and their therapeutic potential in disease settings are currently under investigation [191,334]. A chemical compound 5-(4-methoxyarylimino)-2-N-(3,4-dichlorophenyl)-3-oxo-1,2,4-thiadiazolidine (P(3)-25), which possesses anti-bacterial and anti-fungal activities, specifically inhibits TRAF2-mediated NF-κB activation while enhancing TRAF2-mediated AP-1 activation [335]. However, the diverse and cell type-specific functions of TRAFs may prevent systemic administration of therapeutic agents that directly target TRAFs, and local or cell-specific drug delivery needs to be exercised. Alternatively, therapeutic strategies may be designed to specifically manipulate TRAF-interacting partners or downstream signaling pathways. For example, pharmacological inhibitors for cIAP1/2 are currently at various stages of clinical trials for cancers [107], and may be applied to other TRAF-mediated diseases too. Further in-depth understanding of TRAF signaling pathways will serve as experimental framework to be translated into such therapeutic development.

## Competing interests

The author declares that she has no competing financial interests.
